# Cardiovascular magnetic resonance in pregnancy: Insights from the cardiac hemodynamic imaging and remodeling in pregnancy (CHIRP) study

**DOI:** 10.1186/1532-429X-16-1

**Published:** 2014-01-03

**Authors:** Robin A Ducas, Jason E Elliott, Steven F Melnyk, Sheena Premecz, Megan daSilva, Kelby Cleverley, Piotr Wtorek, G Scott Mackenzie, Michael E Helewa, Davinder S Jassal

**Affiliations:** 1 Section of Cardiology, Department of Internal Medicine, Faculty of Medicine, University of Manitoba, Rm Y3531, Bergen Cardiac Care Centre, St. Boniface General Hospital, 409 Tache Avenue, Winnipeg, Manitoba R2H 2A6, Canada; 2 Department of Obstetrics, Gynecology & Reproductive Sciences, Faculty of Medicine, University of Manitoba, Winnipeg, Manitoba, Canada; 3 Institute of Cardiovascular Sciences, St. Boniface Research Centre, University of Manitoba, Winnipeg, Manitoba, Canada; 4 Section of Cardiac Anesthesia, Department of Anesthesia, Faculty of Medicine, University of Manitoba, Winnipeg, Manitoba, Canada; 5 Department of Radiology, Faculty of Medicine, University of Manitoba, Winnipeg, Manitoba, Canada

**Keywords:** Pregnancy, Cardiovascular magnetic resonance, Cardiovascular remodeling, Transthoracic echocardiography

## Abstract

**Background:**

Cardiovascular disease in pregnancy is the leading cause of maternal mortality in North America. Although transthoracic echocardiography (TTE) is the most widely used imaging modality for the assessment of cardiovascular function during pregnancy, little is known on the role of cardiovascular magnetic resonance (CMR). The objective of the Cardiac Hemodynamic Imaging and Remodeling in Pregnancy (CHIRP) study was to compare TTE and CMR in the non-invasive assessment of maternal cardiac remodeling during the peripartum period.

**Methods:**

Between 2010–2012, healthy pregnant women aged 18 to 35 years were prospectively enrolled. All women underwent TTE and CMR during the third trimester and at least 3 months postpartum (surrogate for non-pregnant state).

**Results:**

The study population included a total of 34 women (mean age 29 ± 3 years). During the third trimester, TTE and CMR demonstrated an increase in left ventricular end-diastolic volume from 95 ± 11 mL to 115 ± 14 mL and 98 ± 6 mL to 125 ± 5 mL, respectively (p < 0.05). By TTE and CMR, there was also an increase in left ventricular (LV) mass during pregnancy from 111 ± 10 g to 163 ± 11 g and 121 ± 5 g to 179 ± 5 g, respectively (p < 0.05). Although there was good correlation between both imaging modalities for LV mass, stroke volume, and cardiac output, the values were consistently underestimated by TTE.

**Conclusion:**

This CMR study provides reference values for cardiac indices during normal pregnancy and the postpartum state.

## Background

Cardiovascular disease in pregnancy is the leading cause of maternal morbidity and mortality in North America [1,2]. In the developed world, hypertension, arrhythmias, valvular heart disease, heart failure, and other acquired diseases of the maternal circulation can severely impact pregnancies [1]. A growing number of women with pre-existing congenital heart disease also require cardiac consultation and imaging as part of their pre-conception planning [3]. As the hemodynamic changes of pregnancy may be poorly tolerated in a woman with pre-existing heart disease, adequate evaluation of a woman’s cardiac status using cardiac imaging is often necessary to ensure optimal maternal and fetal outcomes.

The maternal cardiovascular system is subject to a reversible series of structural and functional adaptations during pregnancy. While these changes are normally well tolerated in healthy women, they can present a serious challenge to pregnant women with underlying cardiovascular disease. Driving these normal hemodynamic changes of pregnancy is a fall in systemic vascular resistance (SVR), resulting from peripheral vasodilation and a low-resistance utero-placental circulation [4-6]. Mean arterial pressure falls during the first half of pregnancy, then rises to pre-pregnant levels by term. Plasma volume increases by up to 50% as a result of avid salt and water retention [7,8]. The combination of decreased left ventricular (LV) afterload, increased preload, and a concomitant increase in resting heart rate leads to an increase in cardiac output (CO) over the same period [4,9-16]. Varying degrees and patterns of secondary atrial enlargement and ventricular hypertrophy have been described during pregnancy, along with increases in the cross-sectional areas of the mitral, pulmonic, and tricuspid valves [17]. In the postpartum period, the physiologic adaptations of pregnancy undergo reversal to their pre-pregnant state, mostly occurring shortly after delivery [15].

Various diagnostic imaging methods have been used to characterize the hemodynamic and structural adaptations of the maternal cardiovascular system during and after pregnancy. These methods range from the invasive dye-dilution technique in 1915 [18], the Cournand cardiac catheterization technique with the direct Fick method in the late 1940s [19,20], M-mode echocardiography beginning in the 1960s and refined with pulsed-wave Doppler [21], and more recently, Swan-Ganz pulmonary artery catheterization [22]. Currently, transthoracic echocardiography (TTE) is the most commonly used non-invasive cardiac imaging modality during pregnancy. Transthoracic echocardiography has several advantages as an imaging modality including its widespread availability, portability, excellent temporal resolution, and lack of radiation exposure. However, TTE has a number of notable limitations, including moderate intra- and inter-observer variability [23], use of geometric assumptions that may not accurately describe the maternal heart during pregnancy [23,24], and poor image quality in individuals with increased body habitus or poor acoustic windows.

In contrast, cardiovascular magnetic resonance (CMR) is highly reproducible and accurate in the determination of cardiac volumes, and is not limited by variations in ventricular geometry, body habitus, or exposure to ionizing radiation [25,26]. CMR is considered to be a safe imaging modality in pregnancy for both the mother and fetus [27,28]. There have been no harmful effects found of in utero exposure to CMR in children up to 9 years of follow-up [29,30]. CMR has recently been utilized in the clinical management of aortic dissection, peripartum cardiomyopathy, congenital valvular lesions and other maternal cardiac disease states during pregnancy and the postpartum period [31-39]. However, the role of CMR in the non-invasive assessment of maternal cardiac adaptation during normal, healthy pregnancies remains ill defined. Establishing reliable structural and hemodynamic CMR reference values for normal pregnancies is of benefit in interpreting abnormal changes observed in maternal cardiovascular disease states, as well as in the management of pregnant patients with pre-existing cardiac disease.

The objective of the Cardiac Hemodynamic Imaging and Remodeling in Pregnancy (CHIRP) study was to compare TTE and CMR in the non-invasive assessment of physiologic maternal cardiac remodeling during the peripartum period.

## Methods

### Study population

This was a prospective cohort study (November 1, 2010-September 1, 2012 inclusive) of healthy volunteers recruited from prenatal obstetric clinics at two tertiary care centres. Eligible subjects were between the ages of 18 and 35 years old at their last normal menstrual period, carrying a healthy singleton pregnancy, and had no previous pregnancy carried beyond 14 weeks gestational age. Exclusion criteria included multiple gestation; any history of cardiac disease or cardiac surgery; any history of thyroid disease, hypertension or diabetes; any significant pregnancy-related complications including gestational diabetes, gestational hypertension, or pre-eclampsia; and/or the presence of pacemakers, surgical clips or other contraindications to CMR. Cardiac imaging by both TTE and CMR was performed in all patients at two time points: initially in the third trimester and then again approximately three months postpartum. Postpartum imaging was performed as a surrogate for the baseline non-pregnant cardiovascular state and used for comparison with third trimester imaging data. The University of Manitoba Human Research Ethics Board approved the study protocol and informed consent was obtained from all patients.

### Echocardiography

Standard TTE (Vivid 7, GE Healthcare, Milwaukee, WI, US) with a multi-frequency transducer and tissue Doppler imaging (TDI) capability was performed in all patients. All TTE scans were performed with women in the left lateral decubitus position to minimize aorto-caval compression and improve patient comfort in the third trimester. The parasternal long axis view was used to obtain cardiac dimensions for the LV, right ventricle (RV), interventricular septum (IVS), posterior wall thickness (PWT), left atrium (LA) and ascending aorta. The apical four chamber and two chamber views were used to obtain LV end systolic volume (LVESV) and LV end diastolic volume (LVEDV) by manually tracing the endocardium. These measurements allowed for calculation of the LV ejection fraction (LVEF) and stroke volume (SV) using the biplane modified Simpson’s method [40]. Left and right atrial volumes (LA and RA) were calculated in the apical four chamber view at end-systole using the area length method. The calculation of LV mass using LV linear dimensions was performed according to the American Society of Echocardiography recommendations [40]. Diastolic function was evaluated using pulsed wave Doppler obtained at the level of the mitral valve, assessing peak early (E) and late (A) mitral inflow velocities, isovolumetric relaxation time (IVRT) and deceleration time (DT). The E/A ratio was calculated as a marker of diastolic function. Tissue Doppler measurements of systolic (S’), early (E’) and late (A’) tissue velocities were obtained at the medial and lateral mitral annulus. The dimensionless E/E’ ratio was calculated as a non-invasive measure of LV filling pressure [41]. Two observers (RD and DJ), blinded to the clinical data, analyzed the echocardiographic images offline using EchoPAC PC (version 110.0.0, GE Healthcare, Milwaukee, WI, US).

### Cardiovascular magnetic resonance

The serial CMR scans were performed using a 1.5 Tesla Siemens Scanner (Sonata, Magnetom, Siemens, Erlangen, Germany). Breath-hold cine imaging was performed using a segmented TrueFISP sequence (temporal resolution 25 ms, slice thickness 8 mm, interslice gap 4 mm, spatial resolution 117 × 192, field of view 360 mm, and GRAPPA parallel imaging) with ECG gating to obtain images representative of the entire cardiac cycle in both long and short axis views. A combination of a 32 channel spine matrix and 6 channel body matrix coil was used. All patients were imaged in a half left lateral decubitus position to minimize aorto-caval compression and improve patient comfort in the third trimester. There were no sedative medications or contrast agents used during this study. Chamber measurements were obtained from the three and four chamber long axis views. Measurement of the aortic sinus, sinotubular junction, and ascending aorta were performed in both the three-chamber and coronal views. Endocardial and epicardial contours were drawn manually for the LV and RV, respectively, at end-systole and end-diastole in each data set with the most basal short axis slice identified as the image which contains at least 50% of circumferential myocardium. Papillary muscles and trabeculations were included in LV and RV mass calculation. Two observers (RD and DJ), blinded to the clinical data, analyzed the CMR images offline using CMR analysis suite (version 3.4.0, Circle Cardiovascular Imaging, Calgary, Alberta, Canada).

### Statistical analysis

All parametric data was reported as mean ± standard deviation (SD). Categorical data was reported as “n” (percentage). Comparison of non-pregnant and third trimester means for each imaging modality was performed using a paired Student’s t-test. Linear regression analyses were performed to assess the correlation between TTE and CMR for CO, stroke volume (SV) and LV mass. Bland-Altman plots of difference versus mean were performed for CO, SV and LV mass with 95% agreement limits of ± 1.96 SD. The Mann–Whitney U test was used to measure the intra and inter-observer variability for LVEDV and LV mass for both imaging modalities. Statistical significance was defined as p < 0.05. SAS version 8.01 (SAS Institute Inc., Cary, NC, US) was used to perform the analysis.

## Results

### Study population

The baseline clinical characteristics of the study population (n = 34) are summarized in Table 1. The mean maternal age at the last normal menstrual period was 29 ± 3 years with a mean pre-pregnancy BMI of 24 ± 4 kg/m^2^. The mean gestational age at third trimester imaging was 237 ± 16 days (34 weeks ± 16 days) and mean number of days for postpartum imaging was 107 ± 25 days (16 weeks ± 25 days).

**Table 1:** Clinical characteristics of study population (n = 34)

**Maternal age at LNMP,***years*	29 ± 3
**Pre-pregnancy BMI,***kg/m*^*2*^	24 ± 4
**Pre-pregnancy BSA,***m*^*2*^	1.7 ± 0.2
**Baseline BP (systolic),***mmHg*	112 ± 10
**Baseline BP (diastolic),***mmHg*	68 ± 8
**Vaginal delivery,***n (%)*	26 (77%)
**Birth weight,***g*	3269 ± 400
**GA at delivery,***days*	278 ± 9
**GA at third trimester imaging** ***,*** *days*	237 ± 16
**Days postpartum at baseline imaging,***days*	107 ± 25

Data are expressed as mean ± SD or number (percentage). *BSA,* body surface area; *BMI,* body mass index; *BP,* blood pressure; *GA*, gestational age; *LNMP,* last normal menstrual period.

### Left ventricular geometry and systolic function

The structural and systolic functional parameters for both imaging modalities in the third trimester and postpartum (baseline) settings are summarized in Table 2. There was a statistically significant increase in LV end diastolic diameter (LVEDD) and LVEDV in the third trimester using both TTE and CMR, with narrower confidence intervals using CMR. Both TTE and CMR demonstrated a statistically significant increase in LV mass in the third trimester. By TTE, there was an increase in LV mass from 111 ± 10 g to 163 ± 11 g, representing a 47% increase above baseline values. Similarly, CMR demonstrated a 48% increase in LV mass over the same period, from 121 ± 5 g to 179 ± 5 g, with tighter confidence intervals. The increase in heart rate (HR) and SV between baseline and the third trimester observed with both imaging modalities, resulted in an appropriate increase in CO during pregnancy. Using TTE and CMR, there was an increase in CO from 3.5 ± 0.8 L/min to 6.3 ± 0.7 L/min and from 4.0 ± 0.3 L/min to 7.4 ± 0.4 L/min, respectively (*p* < 0.05) (Table 2).

**Table 2:** Left and right ventricular parameters by TTE and CMR at baseline (postpartum) and the third trimester in total population (n = 34)

	**TTE**			**CMR**		
	**Baseline**	**Third trimester**	***P***	**Baseline**	**Third trimester**	***P***
	**(postpartum)**			**(postpartum)**		
**LV parameters**						
LVEDD *(mm)*	45 ± 4	52 ± 3*	<0.05	46 ± 1	55 ± 2*	<0.05
LVESD *(mm)*	31 ± 2	33 ± 4	0.81	32 ± 2	32 ± 3	0.85
LVEDV *(mL)*	94 ± 10	114 ± 12*	<0.05	99 ± 6	128 ± 5*	<0.05
LVESV *(mL)*	30 ± 7	30 ± 9	0.83	32 ± 4	33 ± 6	0.81
IVS *(mm)*	9 ± 1	9 ± 2	0.92	8 ± 1	8 ± 1	1.00
PWT *(mm)*	9 ± 1	9 ± 1	1.00	8 ± 1	8 ± 1	1.00
SV *(mL)*	62 ± 12	85 ± 8*	<0.05	68 ± 7	97 ± 6*	<0.05
HR *(bpm)*	60 ± 12	74 ± 9*	<0.05	62 ± 8	79 ± 4*	<0.05
CO *(L/min)*	3.5 ± 0.8	6.3 ± 0.7*	<0.05	4.0 ± 0.3	7.4 ± 0.4*	<0.05
LVEF *(%)*	68 ± 7	70 ± 8	0.68	62 ± 4	63 ± 6	0.42
LV mass *(g)*	111 ± 10	163 ± 11*	<0.05	121 ± 5	179 ± 5*	<0.05
**RV parameters**						
RVEDD *(mm)*	32 ± 6	39 ± 3*	<0.05	33 ± 4	39 ± 3*	<0.05
RVEDV	-	-	-	93 ± 4	115 ± 4*	<0.05
RVEF *(%)*	-	-	-	62 ± 3	61 ± 3	0.82
RV mass *(g)*	-	-	-	51 ± 5	71 ± 6*	<0.05
RV FAC *(%)*	43 ± 6	44 ± 5	0.81	-	-	-
TAPSE *(cm)*	3.0 ± 0.2	3.1 ± 0.4	0.76	-	-	-
PASP *(mmHg)*	25 ± 4	30 ± 5	0.67	-	-	-

Data are expressed as mean ± SD. *CO*, cardiac output; *HR,* heart rate; *IVS*, interventricular septum; *LV,* left ventricle; *LVEDD*, left ventricular end-diastolic diameter; *LVEF*, LV ejection fraction; *LVEDV*, LV end-diastolic volume; *LVESD*, LV end-systolic diameter; *LVESV*, LV end-systolic volume; *PASP,* pulmonary artery systolic pressure; *PWT*, posterior wall thickness; *RV,* right ventricle; *RVEDD*, right ventricular end-diastolic diameter; *RVEF,* RV ejection fraction; RV *FAC*, fractional area change; *SV*, stroke volume; *TAPSE,* tricuspid annular plane systolic excursion; *P < 0.05, third trimester vs. baseline within each imaging modality.

**Figure 1:**
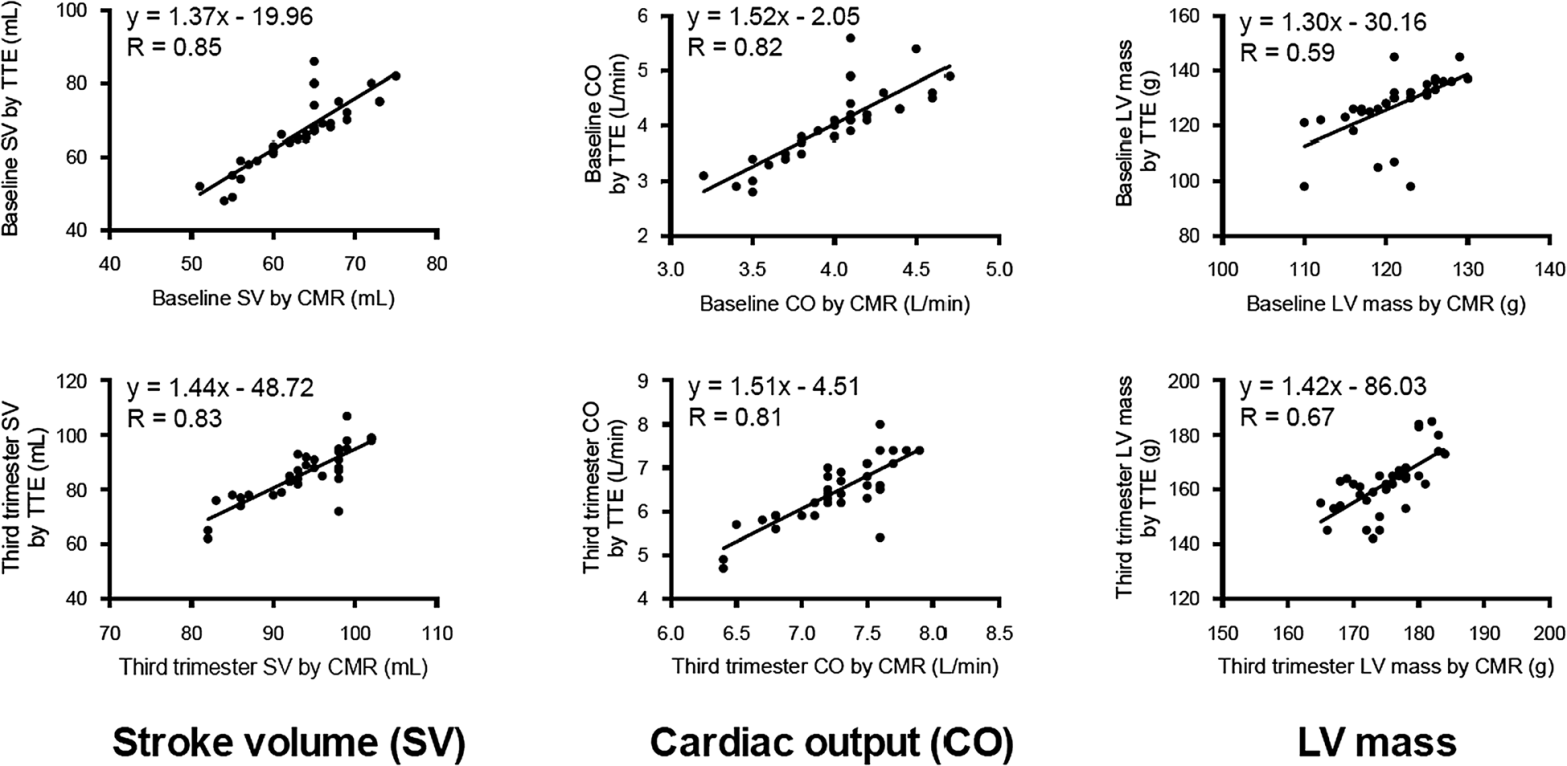
Linear regression plots demonstrating good correlation between TTE and CMR for the assessment of stroke volume (SV), cardiac output (CO) and left ventricular (LV) mass at baseline and in the third trimester.

**Figure 2:**
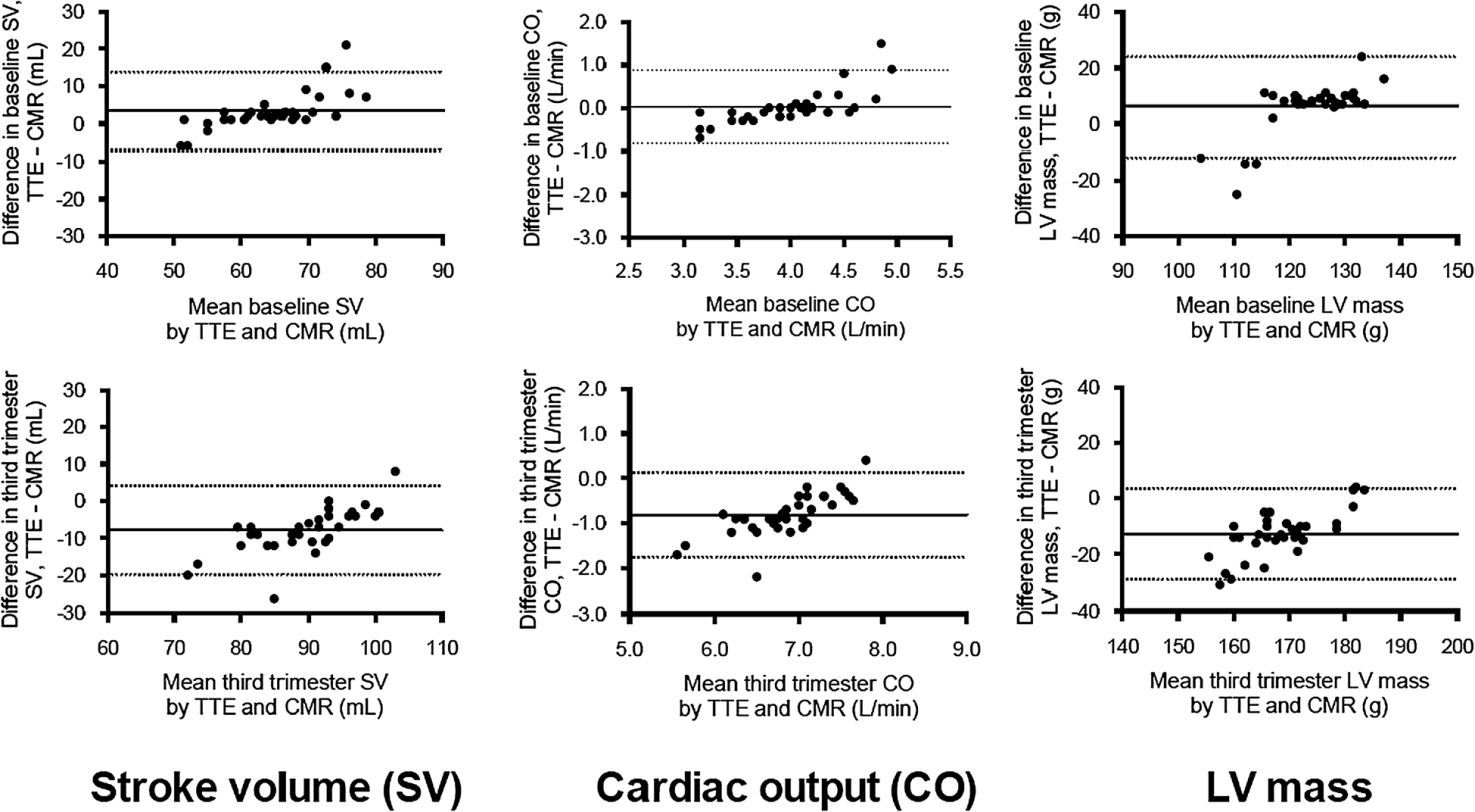
**Bland-Altman plots comparing TTE and CMR assessments of stroke volume (SV), cardiac output (CO) and left ventricular (LV) mass in baseline and third trimester states.** Plots demonstrate TTE tending to underestimate the parameters as compared to CMR. Solid lines indicate mean difference (bias); dotted lines indicate 95% confidence intervals.

Linear regression analysis for SV, CO and LV mass demonstrated good correlation between both TTE and CMR at baseline and in the third trimester as shown in Figure 1. The baseline R-values for SV, CO and LV mass were 0.85, 0.82 and 0.59, respectively. In the third trimester, the R-values for SV, CO and LV mass were 0.83, 0.81 and 0.67, respectively. The Bland-Altman plots for the same parameters are shown in Figure 2. At both time points, TTE showed a slightly negative bias on the Bland-Altman plots in the assessment of SV, CO and LV mass as compared to CMR.

### Right ventricular geometry and systolic function

The RV end diastolic diameter (RVEDD) increased significantly from the non-pregnant state to the third trimester, as determined by both TTE (32 ± 5 mm to 39 ± 3 mm) and by CMR (33 ± 4 mm to 39 ± 3 mm) (Table 2). RV mass as analyzed by CMR increased from a mean of 51 ± 5 g at baseline to 71 ± 6 g in the third trimester (*p* < 0.05), while RV volume increased from 93 ± 4 mL to 115 ± 4 mL (*p* < 0.05). There was no change in the RV systolic function as measured by TTE or CMR.

### Atrial and aortic root geometry

Both LA and RA enlargement was observed from the non-pregnant state to the third trimester, using TTE and CMR (Table 3). The LA end systolic volume measured by CMR increased by approximately 50% from 32 ± 3 mL to 48 ± 3 mL in the third trimester. Similarly, the RA end systolic volume measured by CMR increased by 55% from 29 ± 2 mL to 45 ± 4 mL. A smaller degree of biatrial enlargement was observed using TTE.

**Table 3:** Atrial and aortic parameters by TTE and CMR at baseline (postpartum) and the third trimester in total population (n = 34)

	**TTE**			**CMR**		
	**Baseline**	**Third trimester**	***P***	**Baseline**	**Third trimester**	***P***
	**(postpartum)**			**(postpartum)**		
**Atrial parameters**						
LA diameter *(mm)*	32 ± 3	38 ± 3*	<0.05	31 ± 3	40 ± 3*	<0.05
LA volume *(mL)*	31 ± 5	43 ± 6*	<0.05	32 ± 3	48 ± 3*	<0.05
RA volume *(mL)*	29 ± 3	39 ± 7*	<0.05	29 ± 2	45 ± 4*	<0.05
**Aortic diameters**						
Sinuses *(mm)*	33 ± 1	33 ± 4	0.81	33 ± 2	32 ± 2	0.81
Sinotubular junction *(mm)*	30 ± 2	30 ± 4	0.75	32 ± 2	30 ± 2	0.79
Ascending aorta *(mm)*	31 ± 3	31 ± 3	0.73	31 ± 2	32 ± 3	0.80

Data are expressed as mean ± SD. *LA*, left atrial; *RA*, right atrial. *P < 0.05, third trimester vs. baseline within each imaging modality.

There was no statistically significant change in aortic root or ascending aortic dimensions demonstrated from non-pregnant to third trimester imaging using either TTE or CMR (Table 3).

### Diastolic function

Diastolic parameters obtained by TTE are outlined in Table 4. There was an increase in trans-mitral A wave velocity from 0.7 ± 0.1 cm/s to 1.3 ± 0.2 cm/s from the non-pregnant state to the third trimester (*p* < 0.05). The A wave increase resulted in a decreased E/A ratio in the third trimester from 1.3 ± 0.1 cm/s to 0.7 ± 0.2 cm/s (*p* < 0.05), consistent with mild diastolic dysfunction. The mitral DT, IVRT, and tissue Doppler parameters (including E/E’) did not change significantly from non-pregnant to third trimester imaging.

**Table 4:** Diastolic TTE parameters at baseline (postpartum) and the third trimester in total population (n = 34)

	**Baseline (postpartum)**	**Third trimester**	***P***
**Diastolic parameters**			
E wave *(cm/s)*	0.9 ± 0.1	0.9 ± 0.2	0.87
A wave *(cm/s)*	0.7 ± 0.1	1.3 ± 0.2*	<0.05
E/A	1.3 ± 0.1	0.7 ± 0.2*	<0.05
DT *(ms)*	221 ± 17	224 ± 15	0.65
IVRT *(ms)*	108 ± 10	107 ± 9	0.76
**TDI parameters (LV)**			
Lateral S’ *(cm/s)*	10.3 ± 0.4	11.2 ± 0.3	0.80
Lateral E’ *(cm/s)*	8.0 ± 0.8	8.2 ± 0.6	0.77
Lateral A’ *(cm/s)*	7.8 ± 0.5	7.9 ± 0.4	0.79
Medial S’ *(cm/s)*	9.8 ± 0.6	9.9 ± 0.5	0.71
Medial E’ *(cm/s)*	8.1 ± 0.7	8.2 ± 0.6	0.82
Medial A’ *(cm/s)*	8.0 ± 0.5	8.1 ± 0.4	0.74
Mean E/E’	11 ± 3	11 ± 2	0.78
**TDI parameters (RV)**			
Lateral S’	9.4 ± 0.3	9.5 ± 0.4	0.77
Lateral E’	8.2 ± 0.4	8.1 ± 0.5	0.79
Lateral A’	7.8 ± 0.4	7.6 ± 0.4	0.80

Data are expressed as mean ± SD. *A,* late diastolic transmitral velocity; *A’,* late diastolic myocardial velocity; *E,* early diastolic transmitral velocity; *E’,* early diastolic myocardial velocity; *DT,* deceleration time; *IVRT*, isovolumic relaxation time; *S’*, systolic myocardial velocity; *TDI*, tissue Doppler imaging. *P < 0.05, third trimester vs. baseline.

### Intra-observer and inter-observer variability

The intra- and inter-observer variability of LVEDV and LV mass using both imaging techniques are shown in Table 5. CMR yielded higher reproducibility of cardiac dimensions compared to TTE.

**Table 5:** Intra-observer and inter-observer variability for LVEDV and LV mass

	**Intra-observer**		**Inter-observer**	
	**Absolute**	**%**	**Absolute**	**%**
**LVEDV *(mL)***				
TTE	11.2 ± 6.3	10.3 ± 4.1	13.1 ± 5.4	9.7 ± 4.2
CMR	7.4 ± 4.2	6.2 ± 3.1	9.3 ± 2.5	6.2 ± 3.0
**LV mass *(g)***				
TTE	13.0 ± 3.5	10.4 ± 5.2	12.1 ± 4.3	10.3 ± 3.8
CMR	7.8 ± 3.2	5.9 ± 2.1	9.0 ± 3.2	6.9 ± 2.4

Absolute values are defined as the population mean ± SD of absolute differences between repeated measurements. % values are defined as the population mean ± SD of absolute differences of repeated measurements normalized by the average of the two repeated measurements. *CMR*, cardiovasculart MR; *LVEDV*, left ventricular end-diastolic volume; *LV mass*, left ventricular mass; *TTE*, transthoracic echocardiography.

## Discussion

The diagnostic utility of an imaging modality lies in the knowledge of normal parameters for each variable studied. Without a clear understanding of what is considered normal, diagnosis of disease is challenging, if not impossible. While normal standards of maternal cardiac structure and function are well established for TTE, based on studies conducted since the 1960’s [10-15,17,18,20,41-47] this is the first study to longitudinally assess normal changes in maternal cardiac function and structure during pregnancy using both TTE and CMR. In the current study, we demonstrated an increase in chamber dimensions, LV mass, SV, and CO by both imaging modalities, with tighter confidence intervals using CMR. There was also evidence of mild diastolic dysfunction in the third trimester, with no change in aortic root or ascending aorta measurements. Although there was good correlation between TTE and CMR for LV mass, SV and CO, the values were consistently underestimated by echocardiography.

### Hemodynamic changes

The maternal cardiovascular system undergoes significant physiologic changes in order to support the developing fetus. Previous studies, using TTE in normal pregnancy, have reported that the HR typically increases by 15-20%, SV increases by 20-25%, and CO increases by 30- 50% [4,9-11,13,15]. In our study, we demonstrated that the HR increased by approximately 20%, SV increased by 40%, and CO increased by 80-85% using TTE and CMR. Although the increase in HR seen in our study was similar to previous studies, we observed a greater increase in SV and CO during pregnancy. Recent improvements in endocardial visualization by both TTE and CMR, as well as differences in study design and imaging time points may account for this finding. To the best of our knowledge, this is the first study to longitudinally assess CO and SV using CMR in pregnancy, which is known to have improved accuracy and reproducibility for assessment of LV volumes [24]. Such findings may have implications in pre-conception counseling and managing women with pre-existing heart disease or myocardial dysfunction in pregnancy.

### Left ventricular dimensions and mass

During pregnancy, total blood volume expansion leads to an increase in ventricular preload and compensatory structural changes in the LV. The increases in LV volume and myocardial mass are physiologic adaptations enabling the maternal heart to support the demands of the developing fetus. Previous studies have reported an increase in LVEDV and LV mass in the third trimester, relative to the non-pregnant state [11,12,14]. In the current study, using both TTE and CMR, we demonstrated an increase in LVEDV of 20-30% with an increase in LV mass of 45-50%, similar to previous studies. Our finding of slightly higher values for LV volume and mass both at baseline and in the third trimester for CMR, as compared to TTE, is a reflection of differing image analysis techniques. Whereas CMR routinely includes papillary muscles in volume and mass analyses, they are excluded in echocardiographic measurements [24,26]. Nonetheless, although TTE consistently underestimated LV volumes and mass during the peripartum period as compared to CMR, there was good correlation between both imaging modalities.

### Right ventricular dimensions and mass

Although RV dilation is an accepted physiologic change in pregnancy, poor visualization of this chamber on TTE and lack of standardized parameters have accounted for the paucity of data examining RV remodeling during pregnancy. Whereas quantitative assessment of the RV using TTE is difficult due to its complex geometry, CMR serves as the gold standard for non-invasive assessment of this chamber due to its higher spatial resolution and low intra- and inter-observer variability [42]. A previous study using TTE demonstrated an increase in RVEDD by 18% during the third trimester [17], which is very similar to the values obtained in our study. Using CMR in our study, we also provide new reference values for an increase in RV volume and mass with preserved RV systolic function during pregnancy. In women with pre-existing congenital cardiac conditions that involve the RV, the peripartum use of CMR may be beneficial in the optimal management of this patient population.

### Atrial geometry and diastolic function during pregnancy

During pregnancy, there is physiological dilatation of both atria due to an increase in the effective circulating blood volume. Similar to previous studies that demonstrated an increase in LA volumes by approximately 30% in the third trimester [10,14,43], our study confirmed LA volume increases of 39% and 50% by TTE and CMR, respectively. Although few studies have examined RA volumes during pregnancy, Campos *et al.* demonstrated a 19% increase in RA dimensions in the third trimester by TTE [17]. Our study demonstrated an increase in RA volume of 34% and 55% by TTE and CMR, respectively. With the physiological increase in both atria during pregnancy, compensatory changes in diastolic function occur as well.

Traditional Doppler techniques have been used to characterize diastolic function in pregnancy. Previous echocardiographic studies have demonstrated a decline in the ratio of passive filling to atrial contraction-related ventricular filling during diastole in pregnant women at term [44-48]. This decrease in E/A ratio that has been postulated to reflect mild diastolic dysfunction during the pregnant state was supported in our current study. As compared to these conventional measures of diastolic dysfunction, TDI parameters are independent of preload and afterload, which are both altered by pregnancy. Our results reflect no significant change in TDI parameters during the peripartum state.

### Aortic changes

Although TTE is limited in its ability to assess aortic root and ascending aorta dimensions, CMR can provide multi-planar reformations of the great vessels allowing for greater visibility and more accurate quantification [49]. A recent comparative study of TTE and CMR in pregnant women with pre-existing aortopathies reported that CMR altered clinical decision-making in up to 50% of the study population [35]. Although the clinical utility of CMR was well reported in this study, no CMR reference parameters for aortic dimensions during a normal healthy pregnancy have been previously been established. An important new finding of our study was to provide longitudinal aortic root measurements during healthy pregnancies using CMR. In our study, we demonstrated that aortic root and ascending aorta measurements remain relatively stable during the peripartum state. This finding may have implications in pre-conception counseling and managing women with pre-existing aortopathies in pregnancy.

### Limitations

There are several limitations to the current study. First, we used the postpartum period as a surrogate for the pre-pregnancy state. This was done for logistical reasons as predicting when and whom to image pre-conception to allow creation of a study cohort is neither cost-effective nor practical. Second, since we did not image pregnant women during the first and second trimesters, the degree of structural and functional change in earlier pregnancy was not determined. We performed imaging in the third trimester as several studies have shown that many hemodynamic changes will be fully evolved by 28 weeks gestation [4,9]. Finally, due to the prolonged length of the CMR examinations for the pregnant women, CMR-based blood flow velocity measurements were not obtained and thus could not be compared to the analogous values measured with TTE.

## Conclusion

This CMR study provides reference values for cardiac indices during normal pregnancy and the postpartum state. Future studies are warranted to evaluate the role of CMR in the assessment of pregnant women with pre-existing cardiac disease and/or peripartum cardiomyopathy.

## Abbreviations

CMR: Cardiovascular magnetic resonance; CO: Cardiac output; BMI: Body mass index; BP: Blood pressure; BSA: Body surface area; DT: Deceleration time; GA: Gestational age; HR: Heart rate; IVS: Interventricular septum; IVRT: Isovolumetric relaxation time; LA: Left atrium; LNMP: Last normal menstrual period; LV: Left ventricle; LVEF: Left ventricular ejection fraction; LVEDD: Left ventricular end diastolic diameter; LVEDV: Left ventricular end diastolic volume; LVESD: Left ventricular end systolic diameter; LVESV: Left ventricular end systolic volume; PASP: Pulmonary artery systolic pressure; PWT: Posterior wall thickness; RA: Right atrium; RV: Right ventricle; RVEDD: Right ventricular end diastolic diameter; RVEDV: Right ventricular end diastolic volume; RV FAC: Right ventricle fractional area change; SV: Stroke volume; SVR: Systemic vascular resistance; TAPSE: Tricuspid annular plane systolic excursion; TDI: Tissue Doppler imaging; TTE: Transthoracic echocardiography.

## Competing interests

The authors declare that they have no competing interests.

## Authors’ contributions

RD, JE, SM, SP, and DJ contributed to the design and enrollment of the study participants. RD, SM, SP, MD, KC, PW, and DJ contributed to the post-processing of the echocardiographic and CMR studies. RD, JE, SM, SP, MD, KC, PW, SM, MH, and DJ contributed to the writing of the manuscript. All authors read and approved the final manuscript.
